# Multimodality Diagnosis and Management of Carcinoid Heart Disease: Outcomes and Long-Term Follow-Up

**DOI:** 10.1016/j.jaccas.2026.107926

**Published:** 2026-04-30

**Authors:** Nicholas Johnson, Samuel Krasner, Andrew Lewis, Nikant Sabharwal

**Affiliations:** a Oxford Heart Centre Department of Cardiology, Oxford University Hospitals, Oxford, United Kingdom; b Radcliffe Department of Medicine, John Radcliffe Hospital, University of Oxford, Oxford, United Kingdom

**Keywords:** anticoagulation, cancer, echocardiography, imaging, nuclear medicine, pulmonic valve, thrombus, tricuspid valve, valve replacement

## Abstract

**Background:**

Carcinoid heart disease (CHD) is the rare but serious complication of a paraneoplastic syndrome caused by carcinoid tumors predominantly affecting right-sided cardiac valves and valvular apparatus, leading to significant valvular dysfunction. Multidisciplinary management is paramount for optimal patient outcomes.

**Case Summary:**

A 50-year-old man with chronic flushing, diarrhea, and weight loss was diagnosed with CHD. Echocardiography demonstrated resultant severe tricuspid and pulmonary valve degeneration. He underwent dual-valve replacement, followed by liver debulking surgery with good initial results. Subsequently early, significant valve degeneration occurred.

**Discussion:**

CHD occurs in up to 50% of patients with cardiac syndrome, and insidious symptom onset means diagnosis often delayed. Early investigation and prompt management are therefore imperative to prevent progression and plan multistaged interventions.

## Visual Summary

**Figure:**
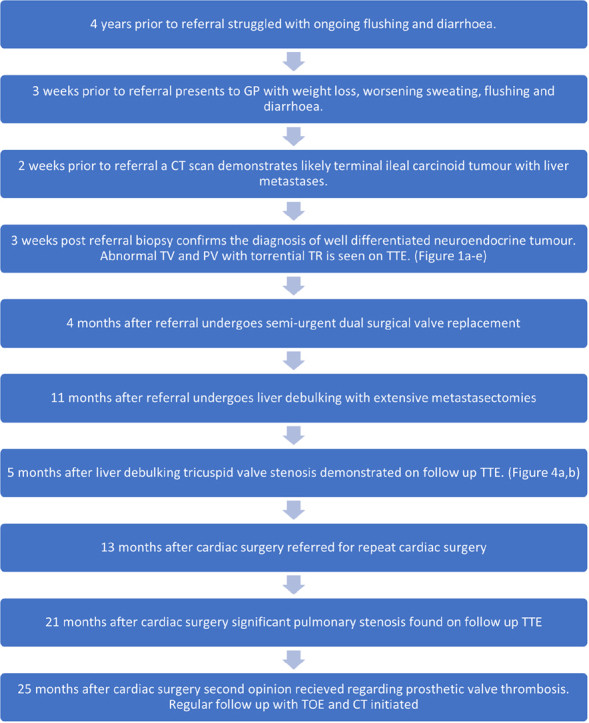

**Visual Summary:**
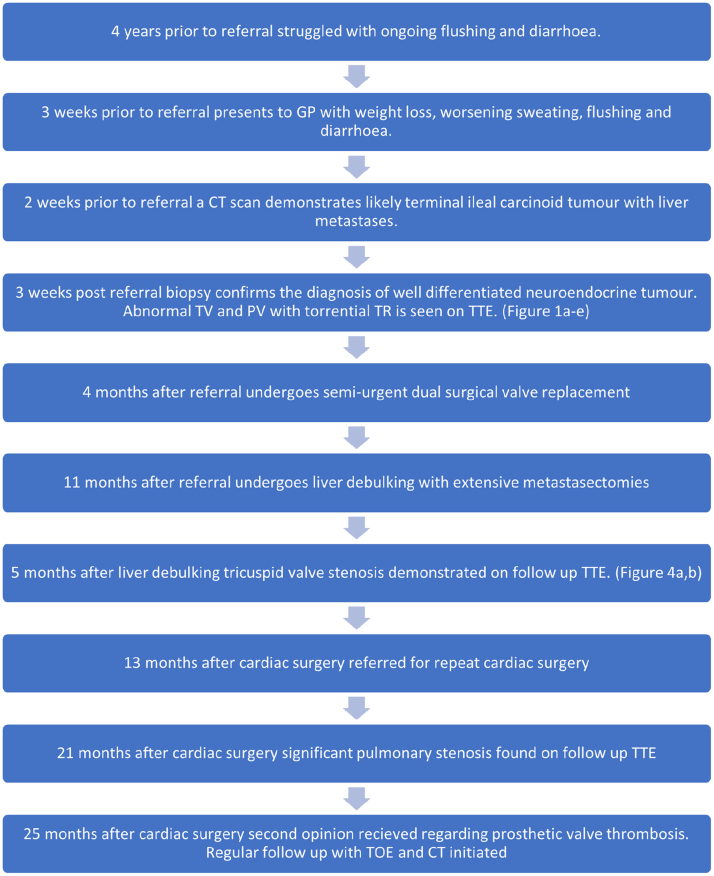
Case Timeline

## History of Presentation

A 50-year-old man presented to primary care with a history of flushing of the face and chest, diarrhea, weight loss, shortness of breath, and ankle swelling. Flushing was the first symptom experienced, occurring after a stressful life episode 4 years prior. Examination demonstrated an elevated jugular venous pressure, a grade 3/6 systolic murmur loudest at the tricuspid area, and bilateral pedal edema rising 3 to 4 inches above the ankles.

**Take-Home Messages**

• Carcinoid syndrome outcomes are improved with early detection and prompt cardiac optimization.
• Multimodality imaging is of significant benefit in diagnosis and follow-up.

## Past Medical History

There was no significant past medical history, and the patient took no regular medications.

## Investigations

He was referred for computed tomography (CT) of the thorax, abdomen, and pelvis. This demonstrated a focal 3-cm avidly enhancing intraluminal lesion within the terminal ileum and 4 hyper-enhancing liver lesions, the largest of which was 10 cm in size.

**Figure 1:**
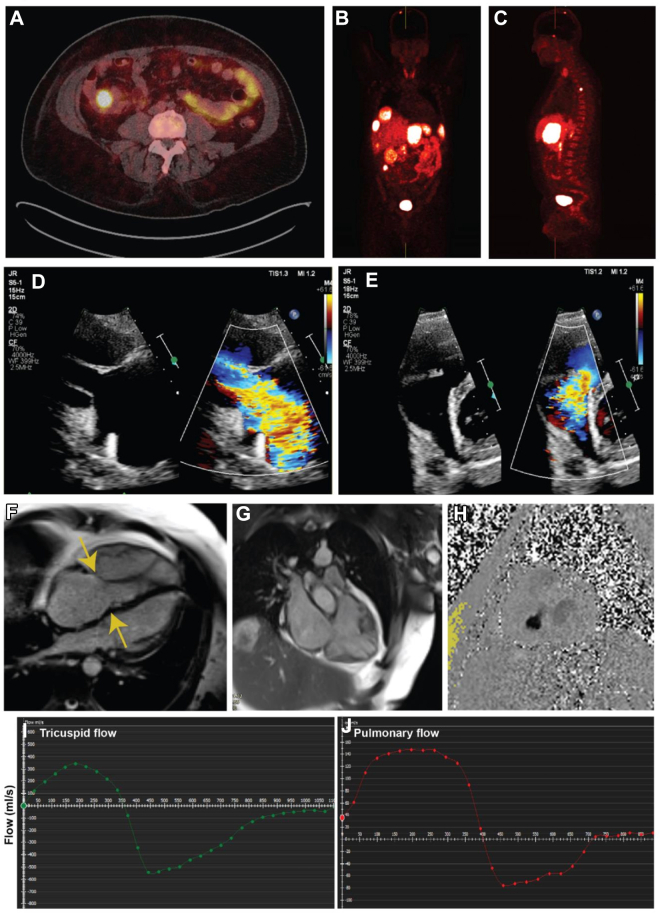
Baseline Characterization of Lesions and Cardiac State (A to C) ^68^Ga-Dotatate PET/CT demonstrating somatostatin positive lesions on axial, coronal, and sagittal planes, respectively. (D) Preoperative TTE demonstrating severe TR (TR jet area: 19 cm^2^, vena contract 23 mm), RA dilation (RA ESA Index 18.7 cm^2^/m^2^) and RV dilation (RV basal diameter 5.1 cm, mid-diameter 4.5 cm, diastolic area 34.4 cm^2^) with thickened and retracted valve leaflets. (E) Preoperative TTE demonstrating a thickened pulmonary valve with mild pulmonary stenosis and regurgitation. (F and G) CMR demonstrating thickening, shortening, and retraction of the tricuspid valve leaflets (F), resulting in a large coaptation defect with severe TR (G). (H) CMR demonstrating thickening of the PV cusps with moderate-severe PV stenosis (valve area 1.0-1.1 cm^2^, peak velocity 2.3 m/s). (I and J) CMR-derived tricuspid and pulmonary transvalve flow velocity curves. CMR = cardiovascular magnetic resonance; ESA = end systolic area; PET/CT = positron emission tomography/computed tomography; PV = pulmonary valve; RA = right atria; RV = right ventricle; TR = tricuspid regurgitation; TTE = transthoracic echocardiography.

He was referred to our tertiary center, where biopsy of the intrahepatic lesions confirmed on histology the diagnosis of a grade 1, well-differentiated, neuroendocrine tumor (NET). ^68^Ga-Dotatate positron emission tomography/CT confirmed the lesions to be somatostatin positive (Figures 1A to 1C) and found avid bone lesions at the T5 and T10 vertebrae and posterior right sixth rib. The patient began treatment with furosemide and subcutaneous lanreotide injections for symptom control.

Transthoracic echocardiography (TTE) demonstrated a grossly dilated right atrium and moderately dilated right ventricle (RV). There was severe tricuspid regurgitation (TR) with thickened and retracted valve leaflets (Figure 1D, Video 1). The pulmonary valve (PV) was thickened, with mild stenosis and regurgitation (Figure 1E, Video 2). Right-heart catheterization demonstrated elevated right-sided pressures, mean right atrial pressure 18 mm Hg, RV pressure 48/0 mm Hg, and pulmonary artery pressure 28/12 mm Hg. Left-heart catheterization demonstrated unobstructed epicardial coronary arteries.

Cardiovascular magnetic resonance (CMR) imaging demonstrated typical findings of carcinoid heart disease (CHD) including thickening, shortening, and retraction of the tricuspid valve (TV) leaflets (Figure 1F), resulting in a large coaptation defect with severe TR (Figure 1G). The PV cusps were also thickened, with moderate-severe PV stenosis (Figure 1H) and mild pulmonary regurgitation (Figure 1I). The RV was moderately dilated (295 mL, 136 mL/m^2^), with preserved systolic function (Right ventricular ejection fraction 58%). Left ventricular function was mildly impaired (LVEF 50%), with no evidence of carcinoid involvement of the left-sided valves.

The case was discussed as part of a multidisciplinary team (MDT) meeting, which recommended to proceed with surgical valve replacement, followed by liver-debulking surgery. The MDT noted the patient's good functional baseline and uneventful prior interventions while on somatostatin analogs. Due to the expected high risk of valve thrombosis, a biological valve prosthesis was the preferred option for valve replacement.

## Management

**Figure 2:**
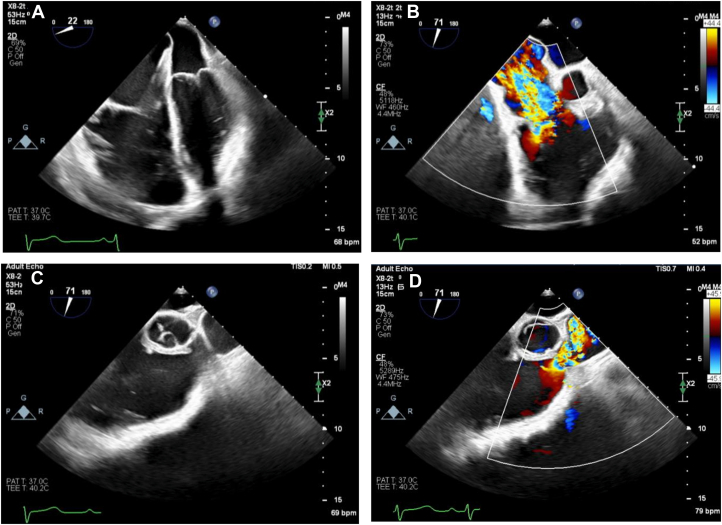
Baseline TOE Imaging at the Time of Cardiac Surgery (A) Grossly dilated right ventricle and right atrium with thickening of the tricuspid valve. Normal-size LV and LA seen. (B) Severe TR seen on baseline intraoperative TOE. (C) RVOT on intraoperative TOE. (D) Demonstration of mild pulmonary stenosis and pulmonary valve thickening on intraoperative TOE. LA = left atria; LV = left ventricle; RVOT = right ventricular outflow tract; TOE = transesophageal echocardiography; TR = tricuspid regurgitation.

**Figure 3:**
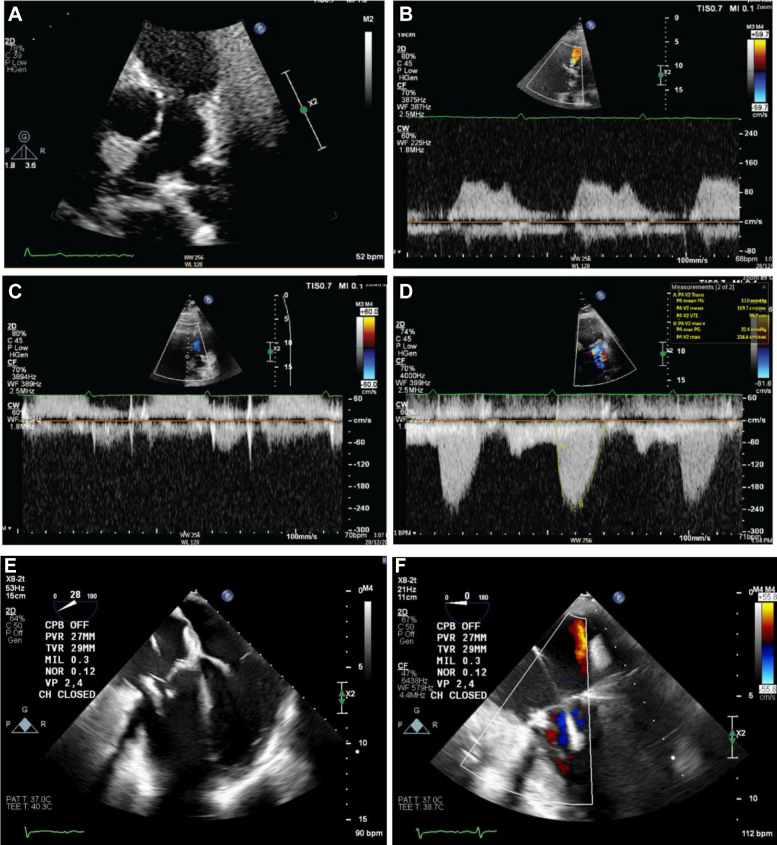
Post-Operative TOE and TTE (A) Intraoperative TOE demonstrating satisfactory pulmonary valve replacement positioning. (B) Postoperative TTE demonstrating tricuspid inflow. (C) Postoperative TTE demonstrating abolition of significant TR gradient (TV mean PG 4 mm Hg). (D) Postoperative TTE demonstrating reduced pulmonary transvalvular gradients (PV max PG 13 mm Hg, mean PG 7 mm Hg). (E) Intraoperative TOE demonstrating satisfactory tricuspid valve replacement positioning. (F) Abolition of significant tricuspid regurgitation as demonstrated on postoperative TOE with color Doppler. PG = pressure gradient; PV = pulmonary valve; TOE = transesophageal echocardiography; TR = tricuspid regurgitation; TTE = transthoracic echocardiography; TV = tricuspid valve.

Alongside lanreotide, oral octreotide was used as required for symptomatic relief. Surgical valve replacement was successfully undertaken with a 27-mm SJM Epic valve sutured into the posterior PV annulus and a 29-mm SJM Epic implanted into the TV annulus with pericardial patch augmentation of main pulmonary artery and RV outflow tracts. Intra-operative transesophageal echocardiogram (TOE) demonstrates successful reduction in TV and PV gradients (Figure 2A to D start of Procedure, Figures 3A and 3E, Video 3 end of procedure). TTE performed 5 days post surgery confirmed both valves were well seated with good function, no significant TR, and reduced valve gradients (Figures 3B to 3D, Video 4). Recovery was uneventful, and the patient was discharged 10 days post surgery on 3 months of warfarin with a target international normalized ratio of 2-3 and bisoprolol.

The Ki-67 score, an indicator of tumor cell activity via histological staining of the protein Ki-67, was 3%. A score >30% is generally considered high. Therefore, the tumor was of intermediate grade. Surgery was not curative but instead for control of carcinoid symptoms and prevention of recurrent valvular degeneration. Cardiopulmonary exercise testing demonstrated generalized deconditioning with a peak power output of 104 W (58% predicted) and peak oxygen consumption of 13.9 mL/kg/min (49% predicted), with cutaneous flushing during exercise.

Liver debulking surgery was planned to occur within 6 months of cardiac surgery to optimize preoperative fitness levels while avoiding repeat degeneration of the cardiac valves. The patient was admitted for liver-debulking surgery 25 weeks post valve replacement. Debulking was undertaken with metastasectomies of the large left-lobe lesion and 2 superficial right-lobe lesions. He was discharged 5 days post operation before readmission due to worsening abdominal pain, where he was managed for a delayed bile leak with endoscopic retrograde cholangiopancreatography and stenting.

## Outcome and Follow-Up

The patient remained symptom free and able to exercise regularly at 3 months. Urinary 5-Hydroxyindoleacetic acid levels normalized, and lanreotide frequency was reduced to every 4 weeks. Primary tumor resection was considered but deferred as symptoms were well controlled, and the primary tumor was small.

**Figure 4:**
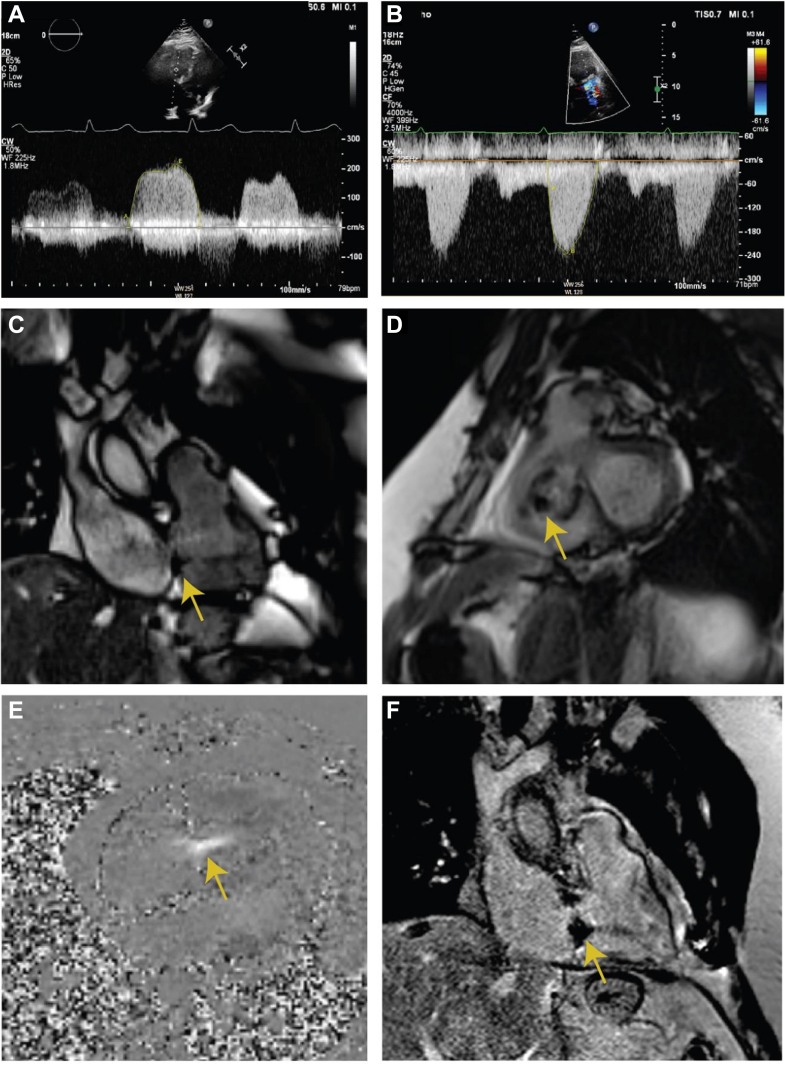
Early Follow-up Imaging (A) TTE of TV inflow showing severe stenosis (mean PG 12.2 mm Hg, max PG 18.6 mm Hg, VTI 86.6 cm). (B) TTE of PV transvalvular gradients remain unchanged at 10-month follow-up. (C and D) CMR demonstrating the 13 × 15-mm structure adherent to the inferior aspect of the TV leaflets, which protrudes into the RV. (E and F) CMR demonstrates no early or late gadolinium uptake. Structure density in keeping with thrombosis. CMR = cardiovascular magnetic resonance; PG = pressure gradient; PV = pulmonary valve; TTE = transthoracic echocardiography; TV = tricuspid valve; RV = right ventricular; VTI = velocity time integral.

Five months following liver debulking, the patient developed recurrent worsening exertional shortness of breath and the sensation of neck swelling. Clinical examination revealed a prominent jugular venous pressure A wave, with TTE demonstrating new significant tricuspid stenosis (Figure 4A). The pulmonary gradient was unchanged (Figure 4B). Given the suboptimal postsurgical transthoracic windows, a further CMR was performed, which confirmed both prostheses were well seated, but the TV leaflets appeared thickened, with a 13 × 15-mm mobile structure adherent to the inferior aspect of the leaflets protruding into the RV (Figures 4C to 4D). The structure had no gadolinium uptake on early or late gadolinium imaging, most in keeping with leaflet thrombus (Figure 4F).

**Figure 5:**
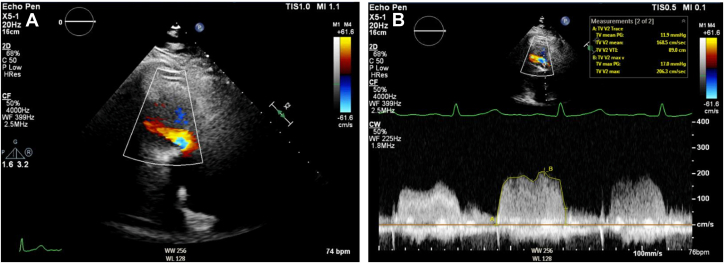
3 Month Follow Up TTE After Initiation of Anticoagulation (A) TTE demonstrating tricuspid inflow with color Doppler jet over the TV. (B) TTE demonstrating no significant improvement in tricuspid inflow velocities despite anticoagulation. TTE = transthoracic echocardiography; TV = tricuspid valve.

The patient began treatment with low-molecular-weight heparin injections (dalteparin 10,000 units twice daily for 7 days) followed by long-term anticoagulation with apixaban. Follow-up 3 months later revealed no improvement in symptoms or tricuspid velocities (Figure 5). On patient consultation, anticoagulation was switched to warfarin with a target international normalized ratio of 3-4. Repeat surgical intervention was considered given his symptoms were having significant impact on quality of life. We rediscussed the case at MDT, and repeat TOE was recommended. TOE confirmed the presence of severe tricuspid stenosis (max PG 13.4 mm Hg, mean pressure gradient 7.3 mm Hg) due to a reduced effective orifice area, secondary to thickening of the TV annulus (Video 5 and 6). The cause was postulated to be due to thrombus or tissue swelling, and we planned for repeat surgical valve replacement due to the patient's young age and good prognosis from carcinoid.

**Figure 6:**
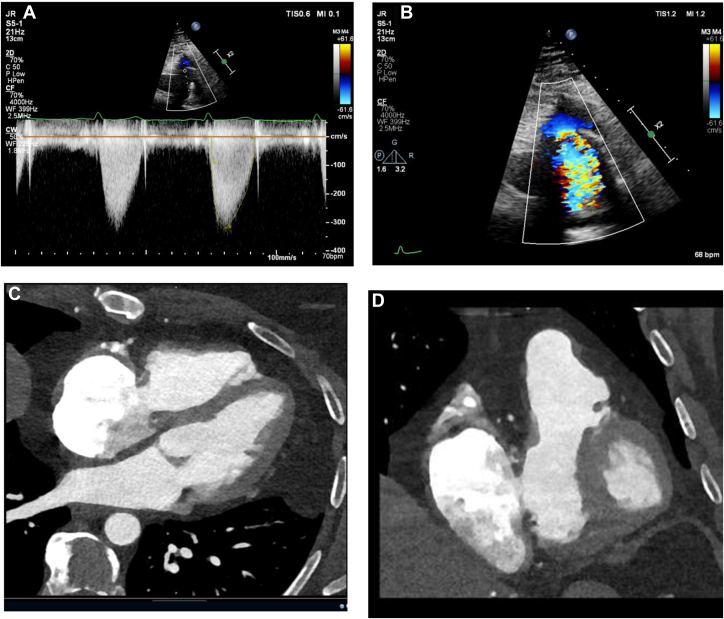
Repeat Surgical Workup (A) TTE of transvalvular PV flow indicating moderate pulmonary stenosis (PA mean PG 25.1 mm Hg, max PG 41.8 mm Hg). (B) TTE color flow Doppler demonstrating flow acceleration across PV. (C and D) Gated CT scan demonstrating likely significant thrombus burden across tricuspid valve. CT = computed tomography; PA = pulmonary artery; PG = pressure gradient; PV = pulmonary valve; TTE = transthoracic echocardiography.

During repeat surgical workup, routine TTE demonstrated new moderate pulmonary stenosis, indicating additional PV degeneration, subsequently confirmed with repeat TOE (Figures 6A and 6B, Video 7). Given evidence of degeneration of both bioprostheses, a second opinion was sought from a high-volume carcinoid center. Consensus opinion remained that both valves had degenerated due to thrombus formation despite warfarinization. There was concern for recurrent NET on the valve prostheses leading to stenosis. However, there were no carcinoid symptoms but normal urinary 5-Hydroxyindoleacetic acid levels; therefore, this was deemed unlikely.

As the patient had no ankle swelling, orthopnea, or paroxysmal nocturnal dyspnea, re-do surgery was postponed, and watchful waiting initiated. His exertional breathlessness may be explained by obesity, with a current body mass index of 39.5. Repeat surgery with significant RV dysfunction on a second run of bypass represents a significant operative mortality with prolonged recovery time, which may not be beneficial given the overall reduced life expectancy seen with NETs. He is undergoing repeat CT scans and/or TOE at 1-year intervals and has been encouraged to lose weight.

## Discussion

Looking back, this case highlights the importance of multimodality imaging and multidisciplinary management in CHD, which is essential given the multiorgan involvement and alteration in cardiac physiology. Carcinoid syndrome is a paraneoplastic syndrome characterized by the release of vasoactive amines and peptides, most commonly serotonin, as a complication of some well-differentiated NETs.^1^ As with our case, carcinoid syndrome most commonly arises from NETs in the gastrointestinal tract.^2^ Upon metastasis to the liver, secreted substances avoid first-pass metabolism and reach the right heart leading to the formation of endocardial plaques, made up of fibroblasts and smooth muscle, on the valves and valvular apparatus. The left heart is normally spared due to pulmonary clearance of vasoactive substances.

Carcinoid tumor diagnostic rates are reportedly increasing.^2^ As seen in this case, symptom onset can be insidious, with diagnosis frequently occurring after liver metastasis has occurred.^3^ Given it is estimated that 50% of those with carcinoid syndrome will develop CHD, early cardiology input is essential.^2^ Current guidelines recommend plasma N-terminal pro-brain natriuretic peptide levels as a screening biomarker for CHD in all patients with carcinoid syndrome and raised urinary 5-Hydroxyindoleacetic acid and/or suspicious symptoms at baseline and follow-up.^4^

Prompt initiation of short- and long-acting somatostatin analogs is essential for symptom control, and trials have demonstrated their ability to prolong progression free survival.^5^ The observed bioprosthetic valve thrombosis (BPVT) is a rare but increasingly recognized cause of early prosthetic valve dysfunction.^6^^,^^7^ Bioprosthetic valves are typically preferred in CHD patients to avoid the need for long-term anticoagulation, which is required with mechanical valves. There is mixed evidence on the degeneration of bioprosthetic valves in CHD, with recent case reports demonstrating rapid valve degeneration, but pooled analysis showing that bioprosthetic valve degeneration remains uncommon.^8^^,^^9^ Increasing TV gradients have shown to be associated with worse survival, and therefore, we would recommend regular imaging follow-up in all CHD patients who have undergone TV intervention.^9^ Confirmed BPVT is usually treated with therapeutic anticoagulation, which frequently results in reduction of gradients and restoration of leaflet motion, although this was not seen in this case; vitamin K antagonists have most clinical experience in this setting.^7^ Current major valve guidelines recommend individualized antithrombotic strategies after bioprosthetic valve implantation and emphasize prompt anticoagulation for suspected/confirmed BPVT while weighing bleeding risk.^10^

## Conclusions

NETs with carcinoid syndrome are rarely curable, and management often involves complex cardiac and abdominal surgery. Cardiac follow-up should be tailored to individual patients, with consideration for type and method of valve intervention used. Multimodality imaging with TTE, TOE, and CMR is complementary for both diagnosis and follow-up to characterize the cardiac disease and effectiveness of interventions.

## Funding Support and Author Disclosures

AL acknowledges funding from the British Heart Foundation (FS/ICRF/24/26111, RE/18/3/34214), the BHF Oxford Centre of Research Excellence (RE/18/3/34214) and the NIHR Oxford Biomedical Research Centre.

## Appendix

Video 1 Initial Transthoracic Echocardiogram at the Parasternal Long-Axis View of the Tricuspid Inflow, Demonstrating Severe Tricuspid Regurgitation and Thickening of Tricuspid Valve Apparatus on Color Doppler

Video 2 Initial Transthoracic Echocardiogram at the Parasternal Short-Axis View Demonstrating Severe Pulmonary Stenosis With Thickening of the Pulmonary Valve Apparatus

Video 3 Postoperative Transthoracic Echocardiogram of the Tricuspid Valve Replacement in the Parasternal Long-Axis, Tricuspid Inflow View Demonstrating Complete Abolishment of the Tricuspid Regurgitation Jet

Video 4 Postoperative Transthoracic Echocardiogram of the Tricuspid Valve Replacement in the Parasternal Short-Axis View

Video 5 Follow-Up Transesophageal Echocardiogram to Characterize the Tricuspid Valve Replacement Demonstrating New Flow Acceleration Due to Severe Tricuspid Stenosis

Video 6 Three-Dimensional Transesophageal Echocardiography of the Tricuspid Valve Replacement Inflow Demonstrating a Severely Decreased Effective Tricuspid Orifice Area

Video 7 Follow-Up Transesophageal Echocardiogram of the Pulmonary Valve Replacement Demonstrating New Moderate Pulmonary Stenosis and Mild Regurgitation
